# Pan‐cancer analysis of oncogenic role of insulin‐like growth factor‐binding proteins and validation in ovarian cancer

**DOI:** 10.1002/cam4.6073

**Published:** 2023-05-18

**Authors:** Wei Tan, Jie Zhang, Zhimin Deng, Fangfang Dai, Lujia Tang, Wei Hu, Hua Liu

**Affiliations:** ^1^ Department of Obstetrics and Gynecology Renmin Hospital of Wuhan University Wuhan China; ^2^ Department of Obstetrics and Gynecology Ultrasound Renmin Hospital of Wuhan University Wuhan China

**Keywords:** cancer hallmark, IGF‐binding proteins, ovarian cancer

## Abstract

**Background:**

Numerous studies have shown that the insulin‐like growth factor (IGF) pathway is highly associated with tumor initial and progression in several tumors. However, compared with IGF1/1R and IGF2/2R, insufficient studies have focused on IGF‐binding proteins (IGFBPs).

**Methods:**

The GDC TCGA and GTEx data of 33 cancers, TCGA pan‐cancer immune phenotypes, tumor mutation burdens, and the copy number alterations of IGFBPs were extracted. Next, the prognostic value of IGFBPs was analyzed based on a univariate Cox analysis. Additionally, the ESTIMATE algorithm was used to calculate stromal and immune scores and tumor purity, and the CIBERSORT algorithm was used to estimate tumor‐infiltrating immunocyte levels. Ultimately, the correlation between IGFBP expression and cancer hallmark pathways was estimated with a Spearman analysis.

**Results:**

The expression of IGFBPs was differentially expressed and correlated with prognosis in specific cancers. IGFBPs may operate as biological markers for carcinogenesis and progression and as prognostic biomarkers. Additionally, IGFBP5 has been proved that promotes the invasion and migration of ovarian cancer.

**Conclusions:**

In general, IGFBPs can serve as predictable biomarkers and potential therapeutic targets for specific tumors. Our results could provide underlying targets for the design of laboratory experiments to elucidate the mechanism of IGFBPs in cancers and identify IGFBP5 as a prognostic factor in ovarian cancers.

## 1 INTRODUCTION

Cancer is a significant risk factor for human longevity and the leading cause of death in the 21st century.^1^ It has always been in the spotlight, due to its high incidence and fatality. Distinct characteristics can be seen in different tumor types, making treatment complex and difficult. Hanahan and Weinberg summarized ten hallmarks of cancer to help us better understand cancer, including genome instability mutations, evading immune destruction, activating invasion and metastasis, and reprogramming energy metabolism were included.^2^ Pan‐cancer analysis can help researchers better identify the similarities and differences between cancers and discover potential targets.^3^

The insulin‐like growth factor (IGF) system plays crucial roles in various physiological and pathological biological processes. This system includes three major ligands (IGF1, IGF2, and insulin), cell surface receptors (the IGF‐I receptor [IGF‐IR], the mannose 6‐phosphate/IGF‐II receptor [M6P/IGF‐IIR], the insulin receptor [IR] and the hybrid IR/IGF‐IR), at least six circulating IGF‐binding proteins (IGFBPs), and ten IGFBP‐related proteases (IGFBP‐rPs).^4^, ^5^, ^6^, ^7^, ^8^ Accumulating evidence has implicated the IGF systems in cancer cell growth, proliferation, metastasis, invasion, apoptosis, and mesenchymal transition.^6^, ^9^, ^10^, ^11^, ^12^, ^13^, ^14^, ^15^ In addition, IGF ligands may serve as potential predictors of some tumors, and the potential of the IGF ligands as therapeutic targets has been evaluated in cancer therapy.^6^, ^16^ Several anti‐IGF‐1R antibodies and two dual IGF1/2 neutralizing antibodies have entered clinical trials.^16^, ^17^ IGFBPs regulate circulating IGF ligand levels by influencing their bioavailability and degradation.^18^ Increasing evidence has shown that IGFBPs have IGF‐independent actions, such as promoting cell apoptosis and inhibiting cell proliferation.^19^, ^20^, ^21^ However, compared with IGF ligands and their cell surface receptors, insufficient studies have focused on IGFBPs. A comprehensive study of IGFBPs is warranted to define their prognostic and therapeutic potential.

In this article, we focused on IGFBP mRNA across cancers. First, the expression of IGFBPs was studied, and the effect of IGFBPs on tumor prognosis was evaluated. Next, we investigated the genetic alteration of IGFBPs. Additionally, the association between IGFBPs and three hallmarks of cancer, evading immune destruction, activating invasion and metastasis, and reprogramming energy metabolism was analyzed. Ultimately, we verified the expression and prognostic effect of IGFBPs in ovarian cancer, and the biologically relevant pathways in ovarian cancer were analyzed. Our results revealed that IGFBPs may be used as prognostic factors for a variety of cancers and play important roles in tumor invasion and metastasis and reprogramming energy metabolism. Validation results in ovarian cancer suggested that IGFBP5 can be a candidate and promising prognostic marker.

## 2 MATERIALS AND METHODS

### 2.1 Data collection

Based on the Cancer Genome Atlas (TCGA) and the Genotype‐Tissue Expression (GTEx) data of 33 cancers, the clinical data of overall survival (OS), disease‐free interval (DFI), progression‐free interval (PFI), disease‐specific survival (DSS) for each TCGA cancer type and TCGA pan‐cancer immune phenotype data were downloaded from the USCS Xena browser (https://xenabrowser.net/datapages/). Although the TCGA project also included the sequencing data of adjacent normal tissue samples from some patients, these “normal” tissue samples may be affected by neighboring tumor cells, which is why GETx samples were applied to match normal tissue.^22^, ^23^ Therefore, we used the “ComBat” function of the “sva” package to correct for the nonbiological batch effects of the standardized RNA‐seq between TCGA and GTEx. The R software was used to extract the expression matrix of IGFBPs. The transformed transcriptional levels were calculated as the log2 of transcripts per million (TPM) +1 and were used in box plots. Next, the tumor mutation burden (TMB) information was obtained from TCGA in the Xena browser. GSE26712 was used to explore the prognostic role of IGFBPs in ovarian cancer. Additionally, we acquired the GSE140082 composed of 380 ovarian cancer samples for external validation from the Gene Expression Omnibus database (GEO, https://www.ncbi.nlm.nih.gov/geo/). Ultimately, the 33 cancers are adrenocortical carcinoma (ACC), bladder urothelial carcinoma (BLCA), breast invasive carcinoma (BRCA), cervical squamous cell carcinoma (CESC), cholangiocarcinoma (CHOL), colon adenocarcinoma (COAD), lymphoid neoplasm diffuse large B cell lymphoma (DLBC), esophageal carcinoma (ESCA), glioblastoma (GBM), brain lower grade glioma (LGG), head and neck squamous cell carcinoma (HNSC), kidney chromophobe (KICH), kidney renal clear cell carcinoma (KIRC), kidney renal papillary cell carcinoma (KIRP), acute myeloid leukemia (LAML), liver hepatocellular carcinoma (LIHC), lung adenocarcinoma (LUAD), lung squamous cell carcinoma (LUSC), mesothelioma (MESO), ovarian serous cystadenocarcinoma (OV), pancreatic adenocarcinoma (PAAD), pheochromocytoma and paraganglioma (PCPG), prostate adenocarcinoma (PRAD), rectum adenocarcinoma (READ), sarcoma (SARC), skin cutaneous melanoma (SKCM), stomach adenocarcinoma (STAD), testicular germ cell tumors (TGCT), thyroid carcinoma (THCA), thymoma (THYM), uterine corpus endometrial carcinoma (UCEC), uterine carcinosarcoma (UCS), and uveal melanoma (UVM).

### 2.2 Possible values of IGF system in survival assays

A univariate Cox proportional risk regression model was used to test the association between IGFBP gene expression and OS, DFI, PFI, and DSS in 33 cancers. Based on the mean expression of IGFBPs, patients were divided into high or low groups in various tumors. In addition, the clinical relevance of IGFBPs across various cancer types was obtained from TIMER2.0 (http://timer.comp‐genomics.org/).^24^ Moreover, the online database Kaplan–Meier (KM) Plotter (https://kmplt.com/analysis/) consisting of GEO, the European Genome‐phenome Archive (EGA, https://www.ebi.ac.uk/ega/), and TCGA was employed to validate the relationship between IGFBPs and the OS of patients affected by 21 distinct types of cancer.^25^

### 2.3 Analysis of the tumor immune microenvironment

The ESTIMATE algorithm was used to calculate stromal and immune scores, as well as tumor purity in tumor microenvironment (TME).^26^ Next, we used the CIBERSORT algorithm to estimate tumor‐infiltrating immunocyte levels.^27^ Both algorithms were based on gene expression profiles. Furthermore, six immune subtypes were identified based on an immunogenomic analysis of TCGA pan‐cancer data.^28^ We then analyzed the expression differences of IGFBPs in distinct subtypes.

### 2.4 Biological pathway analysis

Four tumor‐related biological pathways (DNA repair, EMT, hypoxia, and glycolysis) were downloaded from the molecular signature database (MsigDB) (https://www.gsea‐msigdb.org/gsea/msigdb/index.jsp). Furthermore, we acquired four previously reported metabolic signature gene sets, including amino acid metabolism, carbohydrate metabolism, lipid metabolism, and tricarboxylic acid cycle (TCA).^29^

### 2.5 Validation in ovarian cancer

All patients provided informed consent and were approved by the Ethics Committee of the Renmin Hospital at Wuhan University. Normal ovarian tissue was collected from four patients with benign ovarian tumor. We also obtained ovarian cancer tissue from six patients, and paracancer tissue from two of these patients. The clinical parameters were displayed in Table S1. Total RNA was isolated with TRIzol reagent (Invitrogen). The primers used in the qRT‐PCR analysis are presented in Table S2. Reverse transcription was performed using an mRNA Reverse‐Transcription Kit (Yeasen Biotech Co., Ltd.) according to the manufacturer's instructions. Random primers were added to initiate cDNA synthesis. qRT‐PCR was performed using SYBR Green PCR Mix (Yeasen Biotech Co., Ltd.) on a Bio‐Rad CFX96 system. The 2^−ΔΔCt^ method was performed to calculate the relative gene expression. To explore the changes in the protein levels of IGFBPs, we acquired proteomic data from 25 normal and 100 ovarian cancer tissues from the Clinical Proteomic Tumor Analysis Consortium (CPTAC).^30^ UALCAN is an online site that integrates TCGA transcriptional level data, CPTAC protein level data, and childhood brain tumor data.^31^ It enables researchers to easily analyze the genes of interest in cancer. In this article, we performed UALCAN to present a thorough analysis of IGFBP5 protein expression from CPTAC (http://ualcan.path.uab.edu/analysis‐prot.html).

### 2.6 Cell culture

SKOV3 cells are human serous OC cell lines that were obtained from the American Type Culture Collection (ATCC) cell bank. And the SKOV3 cells were cultured as in our previous results.^32^

### 2.7 Cell invasion assay

SKOV3 without or with IGFBP5 recombinant protein (HY‐P7021, MedChemExpress, MCE) were cultivated in 200 μL of medium without FBS and then placed in the upper chamber with Matrigel‐coated membrane (50 μL; dilution 1:6; Corning Incorporated). Next, 600 μL of 1640 medium containing 10% FBS was introduced into the bottom chamber. After incubation for 24 h, the cells were fixed and stained with crystal violet.

### 2.8 Wound healing assay

SKOV3 cells without or with IGFBP5 recombinant protein were seeded onto 6‐well plates. When the cells covered the entire bottom of the 6‐well plates, the 200 μL pipette tip was used to scratch the adherent cell layer. Subsequently, cells were incubated in an FBS‐free medium for 24 h. Wound healing capacity was monitored via microscopy at 0, and 24 h.

### 2.9 Western blot

Total protein was extracted from cells using protein lysate, which consisted of RIPA buffer (Bioss), PMSF (Beyotime Biotechnology), total protease inhibitor (Servicebio), and phosphatase inhibitor (Servicebio). Next, protein lysates were loaded onto 10% or 12.5% SDS‐polyacrylamide gels (Yeasen Biotechnology Co. Ltd.). Next, protein lysates were transferred to 0.45 mm/0.22 mm polyvinylidene difluoride (Merck Millipore) for 60 min. The membranes were blocked for 1 h at room temperature with 5% nonfat milk. Then, the membranes were incubated with primary antibodies against IGFBP5 (1:500; ABclonal, Cat: A12451), E‐Cadherin (1:50,000; Proteintech, Cat: 20874‐1‐AP), N‐Cadherin (1:4000; Proteintech, Cat: 22018‐1‐AP), MMP9 (1:1000; ABclonal, Cat: A11147), MMP2 (1:1000; ABclonal, Cat: A11144), α‐tubulin (1:7500, ABclonal, Cat: AC012) or GAPDH (1:10,000, ABclonal, Cat: AC036) overnight at 4°C. Subsequently, the membranes were incubated with HRP Goat Anti‐Rabbit IgG solution (1:7500; ABclonal, Cat: AS014) or HRP Goat Anti‐Mouse IgG solution (1:7500; ABclonal, Cat: AS003) for 1 h at room temperature. After washing three times with TBST for 5 min each time, the membranes were covered with ECL reagents (ABclonal). The intensity of protein bands was quantified by Image J software (Ver 1.5.3).

### 2.10 Gene Set Enrichment Analysis (GSEA) of IGFBPs in ovarian cancer

GSEA was conducted to investigate the biological functions in the high‐ and low‐risk groups of each IGFBP with the cut‐off value of IGFBPs. HALLMARK gene sets were downloaded from the MSigDB database (https://www.gsea‐msigdb.org/gsea/msigdb/index.jsp). The top 10 terms of HALLMARK analyses are shown. Gene sets with *p* < 0.05 were considered significantly enriched.

### 2.11 Statistical analysis

The GraphPad_Prism_8 and R software (version 4.2.0) was employed to conduct a source analysis. Using the “limma” R package, gene expression was compared between normal tissue and neoplasms from 25 cancer types with more than five adjacent normal samples or normal tissue samples. Gene expression data from the TCGA and GTEx databases were analyzed using Student's *t*‐test. In addition, the correlations between IGFBPs and the immune scores, immune‐infiltrating level, metabolism pathways, and cancer hallmark pathways were estimated with a Spearman analysis. ANOVA was applied to test the association between gene expression and immune subtypes. *p* < 0.05 was regarded significant.

## 3 RESULTS

### 3.1 Expression landscape of IGFBPs in Pan‐cancer

**FIGURE 1:**
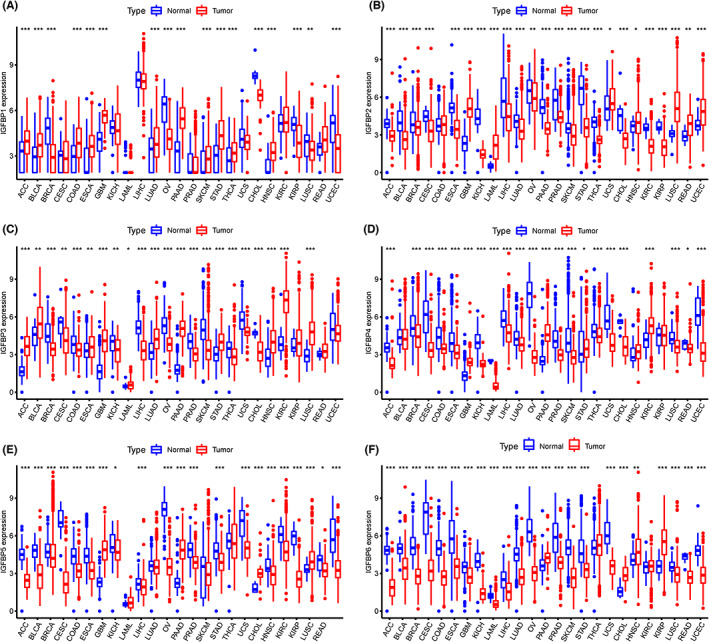
The expression of IGFBPs in pan‐cancer. (A–F) The expression level of IGFBP1 (A), IGFBP2 (B), IGFBP3 (C), IGFBP4 (D), IGFBP5 (E), and IGFBP6 (F) were compared based on the integrated database from TCGA and GTEx. The red and blue boxes represent tumor tissues and normal tissues respectively. *p < 0.05, **p < 0.01, ***p < 0.001. ACC, adrenocortical carcinoma; BLCA, bladder urothelial carcinoma; BRCA, breast invasive carcinoma; CESC, cervical squamous cell carcinoma; CHOL, cholangiocarcinoma; COAD, colon adenocarcinoma; ESCA, esophageal carcinoma; GBM, glioblastoma; HNSC, head and neck squamous cell carcinoma; KICH, kidney chromophobe; KIRC, kidney renal clear cell carcinoma; KIRP, kidney renal papillary cell carcinoma; LAML, acute myeloid leukemia; LIHC, liver hepatocellular carcinoma; LUAD, lung adenocarcinoma; LUSC, lung squamous cell carcinoma; OV, ovarian serous cystadenocarcinoma; PAAD, pancreatic adenocarcinoma; PRAD, prostate adenocarcinoma; READ, rectum adenocarcinoma; SKCM, skin cutaneous melanoma; STAD, stomach adenocarcinoma; THCA, thyroid carcinoma; UCEC, uterine corpus endometrial carcinoma; UCS, uterine carcinosarcoma.

We first matched each solid cancer type in TCGA with the corresponding healthy tissue from the same tissue source in the GTEx project, such as normal ovarian tissue from GTEx and ovarian cancer from TCGA. After the integration of GTEx and TCGA data, the expression levels of IGFBPs in 33 types of tumors were analyzed. The relevant information on the 33 types of cancers is shown in Table S3. Our results revealed that the expression of IGFBP1 was upregulated in ACC, BLCA, COAD, ESCA, GBM, LUAD, PAAD, SKCM, STAD, THCA, HNSC, and KIRP (*p* < 0.05, Figure 1A). Notably, lower IGFBP1 expression was found in sex‐related diseases, such as BRCA, OV, and UCEC (*p* < 0.05, Figure 1A). The expression of IGFBP2 was elevated in GBM, LUSC, READ, and UCEC but downregulated in the other tumors (*p* < 0.05, Figure 1B). The expression trends of IGFBP3 and IGFBP1 in tumors were similar. However, opposite trends in were observed for THCA, COAD, and LUSC, where IGFBP3 was upregulated while IGFBP1 was downregulated (*p* < 0.05, Figure 1C). The expression of IGFBP4/5 was decreased in almost all cancers except for KIRC, PAAD, and GBM (*p* < 0.05, Figure 1D,E). Interestingly, the expression of all IGFBPs was increased in GBM, except for IGFBP6 (*p* < 0.05, Figure 1F). The expression of IGFBP6 was decreased in almost all cancers except for CHOL, HNSC, and KIRP. Notably, the expression of IGFBPs was downregulated in both ovarian and breast cancer but increased in GBM and PAAD.

### 3.2 Effect of IGFBPs expression level on survival

**FIGURE 2:**
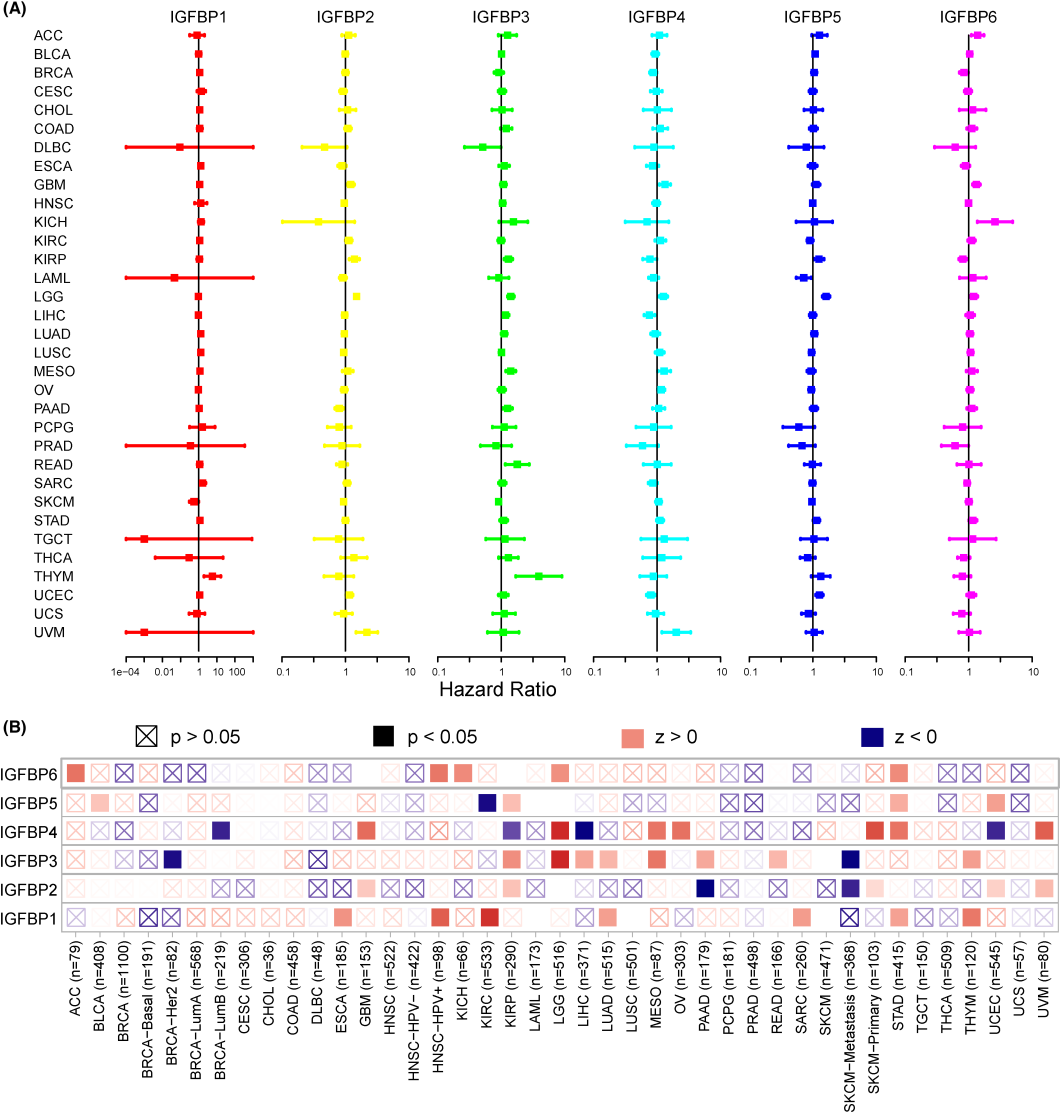
Prognostic analysis of IGFBPs. (A) Forest plot of association of IGFBPs expression with overall survival (OS) in 33 tumors. Different colored lines indicate the risk value of different genes in tumors, hazard ratio < 1 represents low risk, and hazard ratio > 1 represents high risk. (B) The heatmap of IGFBPs across different cancer types based on TIMER2.0 database. ACC, adrenocortical carcinoma; BLCA, bladder urothelial carcinoma; BRCA, breast invasive carcinoma; CESC, cervical squamous cell carcinoma; CHOL, cholangiocarcinoma; COAD, colon adenocarcinoma; DLBC, diffuse large B cell lymphoma; ESCA, esophageal carcinoma; GBM, glioblastoma; HNSC, head and neck squamous cell carcinoma; KICH, kidney chromophobe; KIRC, kidney renal clear cell carcinoma; KIRP, kidney renal papillary cell carcinoma; LAML, acute myeloid leukemia; LGG, lower grade glioma; LIHC, liver hepatocellular carcinoma; LUAD, lung adenocarcinoma; LUSC, lung squamous cell carcinoma; MESO, mesothelioma; OV, ovarian serous cystadenocarcinoma; PAAD, pancreatic adenocarcinoma; PCPG, pheochromocytoma and paraganglioma; PRAD, prostate adenocarcinoma; READ, rectum adenocarcinoma; SARC, sarcoma; SKCM, skin cutaneous melanoma; STAD, stomach adenocarcinoma; TGCT, testicular germ cell tumors; THCA, thyroid carcinoma; THYM, thymoma; UCEC, uterine corpus endometrial carcinoma; UCS, uterine carcinosarcoma; UVM, uveal melanoma.

Univariate Cox proportional hazards model analysis of IGFBPs was used to study the association between the IGFBP expression level and prognosis, and the results are shown in Table S4. As shown in Figure 2A, IGFBP1 expression levels were associated with worse OS in KIRC, LUAD, THYM, SARC, STAD, ESCA, and LUSC (*p* < 0.05). IGFBP2 played a detrimental role in LGG, UVM, KIRP, GBM, UCEC, and KIRC. IGFBP3 was a high‐risk gene in LGG, MESO, KIRP, THYM, READ, PAAD, LIHC, and LUAD (*p* < 0.05). In addition, the increased expression of IGFBP4 predicted worse OS in LGG, GBM, UVM, OV, and MESO and better OS in LIHC, UCEC, BRCA, and KIRP (*p* < 0.05). IGFBP5 had a detrimental effect on LGG, UCEC, KIRP, STAD, and BLCA (*p* < 0.05). IGFBP6 is a low‐risk gene in KIRP, BRCA, and PRAD. By contrast, it is a high‐risk gene in GBM, KICH, ACC, LGG, and STAD (*p* < 0.05). Furthermore, the results of the TIMER 2.0 database were in line with the Cox proportional hazards model. The results revealed that high levels of IGFBP1 were associated with a poor prognosis in ESCA, HNSC, KIRC, LUAD, SARC, STAD, and THYM (*p* < 0.05). IGFBP2 played a detrimental role in KIRP, GBM, UCEC, and UVM but the opposite role in PAAD (*p* < 0.05). A higher level of IGFBP3 correlated with longer survival times in BRCA and SKCM, but predicted a poor prognosis in KIRP, LGG, LIHC, LUAD, MESO, PAAD, READ, and THYM (*p* < 0.05). IGFBP4 was a risk gene in GBM, LGG, MESO, OV, SKCM, STAD, and UVM and a protective factor in UCEC, LIHC, KIRP, and BRCA (*p* < 0.05). The expression of IGFBP5 was linked with poor prognosis in BRCA, KIRP, STAD, and UCEC (*p* < 0.05). IGFBP6 expression was associated with poor prognosis in ACC, HNSC, KICH, LGG, and STAD (*p* < 0.05). Additionally, we applied a Kaplan–Meier plotter based on the GEO, EGA, and TCGA databases to validate the results (Figure S1). The results were consistent with the TIMER 2.0 and Cox proportional hazards models.

OS is widely used as an endpoint for cancers because ambiguity in defining this event is minimal; the patients are either alive or dead. However, longer follow‐up is required to obtain OS data for each patient. Therefore, using OS as an endpoint may weaken a clinical study because death due to causes other than cancer does not necessarily reflect tumor biology, aggressiveness, or responsiveness to therapy.^33^ Therefore, we also conducted Cox risk models using DFI, PFI, and DSS as clinical endpoints. As shown in Figure S2, Table S5, an analysis of DFI data revealed associations between high IGFBP1 expression and poor prognosis in STAD and PRAD (*p* < 0.05). IGFBP2 correlated with worse survival time in LGG and THCA and longer survival time in THCA (*p* < 0.05). The expression of IGFBP3 was linked to worse prognosis in PAAD, LGG, and LIHC (*p* < 0.05). IGFBP4 played a protective role in UCEC (*p* < 0.05). IGFBP5 played a detrimental role in CESC and PAAD and a protective role in SARC (*p* < 0.05). IGFBP6 was a risk gene in ACC and PAAD and a protective factor in KIRP (*p* < 0.05). For DSS and PFI, the results are shown in Tables S6 and S7 and Figure S3. Notably, among the four Cox proportional hazards model analyses of OS, DFI, PFI, and DSS, IGFBP1 was a risk gene in STAD. In LGG, IGFBP2 and IGFBP3 played a detrimental role (*p* < 0.05). Additionally, IGFBP3 was a risk factor in LIHC and IGFBP4 was a protective factor in UCEC (*p* < 0.05). The expression of IGFBP6 was associated with longer survival time in KIRP among four Cox proportional hazards model analyses (*p* < 0.05).

### 3.3 Genetic analysis of the IGFBPs

**FIGURE 3:**
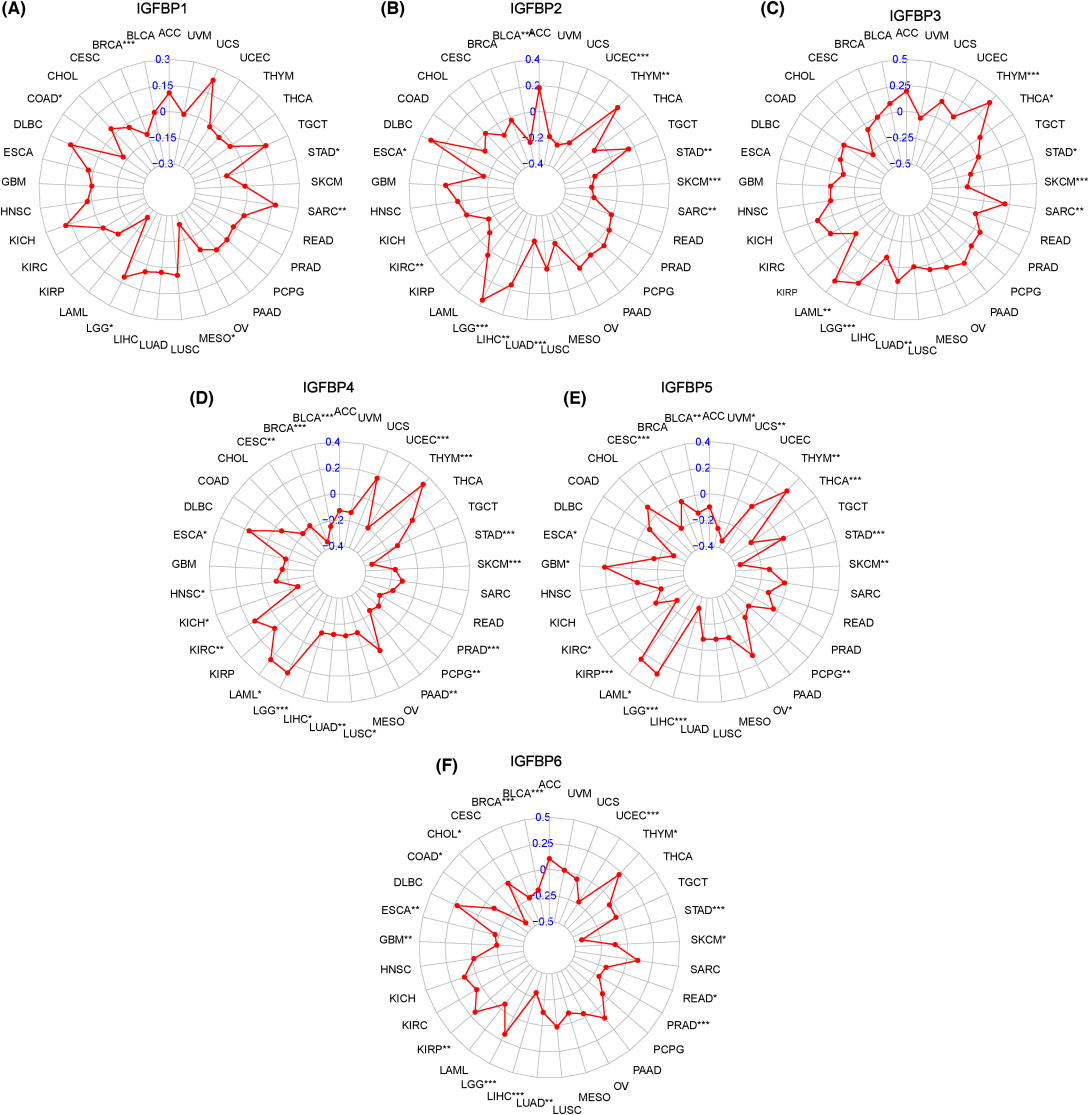
The relationship between the expression of IGFBPs and TMB. (A–F) The radar map displaying the correlation of TMB with IGFBP1 (A), IGFBP2 (B), IGFBP3 (C), IGFBP4 (D), IGFBP5 (E), and IGFBP6 (F). *p < 0.05, **p < 0.01, ***p < 0.001. ACC, adrenocortical carcinoma; BLCA, bladder urothelial carcinoma; BRCA, breast invasive carcinoma; CESC, cervical squamous cell carcinoma; CHOL, cholangiocarcinoma; COAD, colon adenocarcinoma; DLBC, diffuse large B cell lymphoma; ESCA, esophageal carcinoma; GBM, glioblastoma; HNSC, head and neck squamous cell carcinoma; KICH, kidney chromophobe; KIRC, kidney renal clear cell carcinoma; KIRP, kidney renal papillary cell carcinoma; LAML, acute myeloid leukemia; LGG, lower grade glioma; LIHC, liver hepatocellular carcinoma; LUAD, lung adenocarcinoma; LUSC, lung squamous cell carcinoma; MESO, mesothelioma; OV, ovarian serous cystadenocarcinoma; PAAD, pancreatic adenocarcinoma; PCPG, pheochromocytoma and paraganglioma; PRAD, prostate adenocarcinoma; READ, rectum adenocarcinoma; SARC, sarcoma; SKCM, skin cutaneous melanoma; STAD, stomach adenocarcinoma; TGCT, testicular germ cell tumors; THCA, thyroid carcinoma; THYM, thymoma; UCEC, uterine corpus endometrial carcinoma; UCS, uterine carcinosarcoma; UVM, uveal melanoma.

Tumorigenesis is a complex multi‐step process involving the interaction of genetic changes.^34^ Somatic missense mutations strongly contribute to the generation of new tumor epitopes.^35^ TMB is defined as the total number of somatic gene coding errors, base substitutions, and gene insertion or deletion errors detected per million bases.^36^ TMB is an emerging characteristic of cancer and may predict clinical response to immune therapy.^37^ Regarding TMB, the expression of IGFBP1 negatively correlated with TMB in BRCA and MESO but positively correlated with TMB in LGG and SARC (*p* < 0.05, Figure 3A). IGFBP2 expression positively correlated with THYM, LGG, and LIHC but inversely correlated with BLCA, UCEC, STAD, SARC, SKCM, and LUAD (*p* < 0.05, Figure 3B). Furthermore, IGFBP3 expression positively correlated with TMB in THYM, SARC, LAML, and LGG. By contrast, IGFBP3 negatively correlated with TMB in STAD, SKCM, and LUAD (*p* < 0.05, Figure 3C). IGFBP4 positively correlated with TMB in THYM, LGG, and LAML but negatively correlated with TMB in BRCA, CESC, UCEC, BLCA, ESCA, LIHC, LUAD, STAD, SKCM, PRAD, PAAD, PCPG, and LUSC (*p* < 0.05, Figure 3D). The level of IGFBP5 negatively correlated with TMB in OV, CESC, UCS, BLCA, UVM, STAD, SKCM, PCPG, KIRC, KIRP, and LIHC but positively correlated with TMB in LGG, LAML, and THYM (*p* < 0.05, Figure 3E). A positive association was observed between IGFBP6 and TMB in KIRP, LGG, and THYM, whereas an inverse association was observed in BRCA, ESCA, UCEC STAD, SKCM, READ, PRAD, CHOL, LUAD, and BLCA (*p* < 0.05, Figure 3F).

### 3.4 Analysis of immune landscape

Tumor cells can resist immune surveillance and clearance via an immune escape mechanism. Accordingly, the correlations between IGFBPs and immune and stromal scores were evaluated in 33 cancers (Figure S4A,B). Compared with other IGFBPs, the correlation between IGFBP1 and immune score was not strong. Notably, IGFBP3/4/5/6 positively correlated with immune scores in BLCA, COAD, ESCA, GBM, HNSC, LUAD, LUSC, STAD, and OV, whereas IGFBP2 showed an inverse association (*p* < 0.05). Interestingly, the correlation between IGFBPs and stromal scores was consistent with the trend of the immune score. The immune and stromal scores reflect the abundances of the immune infiltrate and stromal cells, respectively, and higher respective scores indicate a larger proportion of the corresponding component in the TME. Tumor purity can reflect the ratio of tumor cells component in TME. Regarding tumor purity, the expression of IGFBPs negatively correlated with tumor purity, except for IGFBP2 in PCPG, KIRP, and OV (*p* < 0.05, Figure S4C).

Tamborero et al. comprehensively analyzed the interactions between tumors and infiltrating immune cell populations and then identified six immunophenotypes, which were composed of Wound Healing (Immune C1), IFN‐gamma Dominant (Immune C2), Inflammatory (Immune C3), Lymphocyte Depleted (Immune C4), Immunologically Quiet (Immune C5), and TGF‐beta Dominant (Immune C6).^38^ A survival analysis of the immunophenotypes showed that the C4 and C6 subtypes had the worst prognosis, whereas the C3 and C5 subtypes had the best prognosis, which was consistent with results previously reported by Zhang et al (Figure S5A).^39^ These subtypes are suggested to be associated with tumor progression. Except for the expression of IGFBP1, the expression levels of almost all IGFBPs were reduced in the C5 subtype and elevated in the C6 subtype, suggesting that these IGFBPs may be associated with poor prognosis (*p* < 0.001, Figure S5B). Next, the correlation between immune cell infiltration and the expression of the corresponding IGFBPs was assessed for each cancer. |cor| > 0.3 and *p* < 0.05 were considered as strong correlation. However, there was no strong association between IGFBPs and the level of immune cells in the tumor microenvironment (Figure S6).

### 3.5 Correlation with tumor biological and metabolism pathways

**FIGURE 4:**
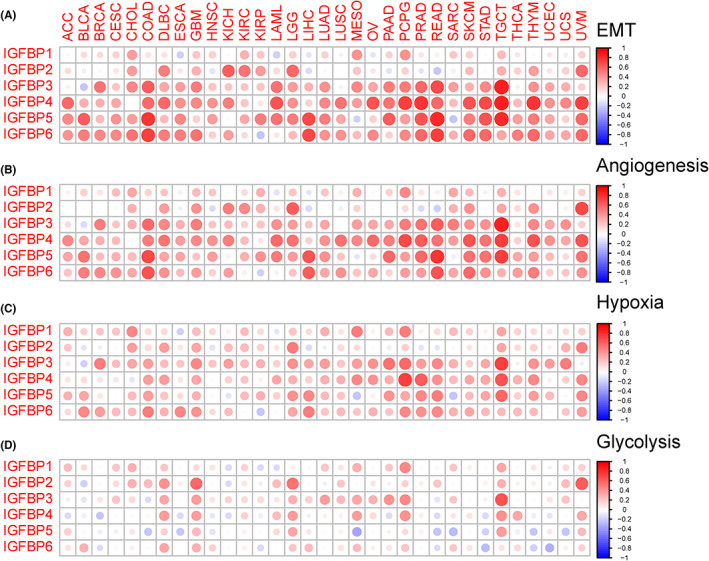
Correlation of tumor invasion and metastasis‐related biology pathway with IGFBPs. The heatmap of the correlation of IGFBPs with epithelial‐mesenchymal transition (A), angiogenesis (B), hypoxia (C), and glycolysis (D). Red dots indicate a positive correlation between gene expression, and blue dots indicate a negative correlation. ACC, adrenocortical carcinoma; BLCA, bladder urothelial carcinoma; BRCA, breast invasive carcinoma; CESC, cervical squamous cell carcinoma; CHOL, cholangiocarcinoma; COAD, colon adenocarcinoma; DLBC, diffuse large B cell lymphoma; ESCA, esophageal carcinoma; GBM, glioblastoma; HNSC, head and neck squamous cell carcinoma; KICH, kidney chromophobe; KIRC, kidney renal clear cell carcinoma; KIRP, kidney renal papillary cell carcinoma; LAML, acute myeloid leukemia; LGG, lower grade glioma; LIHC, liver hepatocellular carcinoma; LUAD, lung adenocarcinoma; LUSC, lung squamous cell carcinoma; MESO, mesothelioma; OV, ovarian serous cystadenocarcinoma; PAAD, pancreatic adenocarcinoma; PCPG, pheochromocytoma and paraganglioma; PRAD, prostate adenocarcinoma; READ, rectum adenocarcinoma; SARC, sarcoma; SKCM, skin cutaneous melanoma; STAD, stomach adenocarcinoma; TGCT, testicular germ cell tumors; THCA, thyroid carcinoma; THYM, thymoma; UCEC, uterine corpus endometrial carcinoma; UCS, uterine carcinosarcoma; UVM, uveal melanoma.

Cancer invasion and metastasis and metabolic reprogramming are two typical hallmarks.^2^ We first explored the association between IGFBPs and tumor biological pathways (EMT, angiogenesis, hypoxia, and glycolysis) related to cancer invasion and metastasis. The results were shown in Figure 4. The correlation between IGFBP1 and EMT pathway was not obvious. However, there was a strongly positive association between IGFBP3/4/5/6 and the EMT, angiogenesis, and hypoxia pathways in OV, READ, PRAD, PCPG, and TGCT (cor > 0.3, *p* < 0.05, Figure 4A–C). For glycolysis, a positive correlation was observed with all IGFBPs in GBM, LGG, and PCPG (*p* < 0.05, Figure 4D).

**FIGURE 5:**
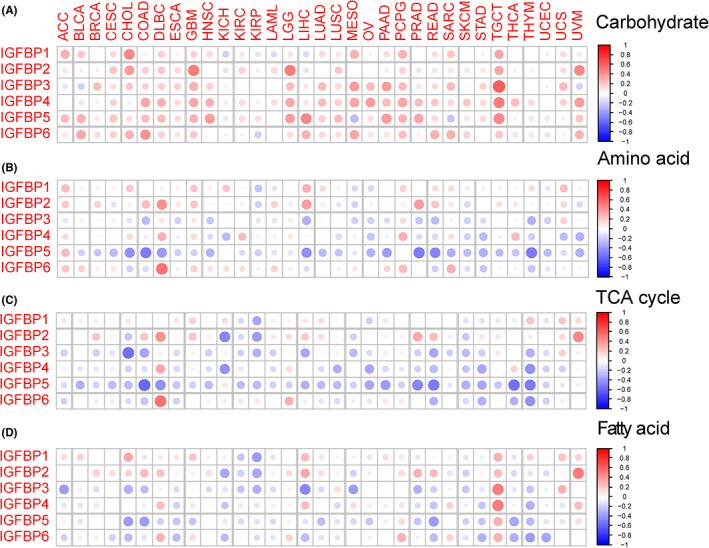
Correlation of tumor metabolism reprogramming with IGFBPs. The heatmap shows the correlation of IGFBPs with carbohydrate (A), amino acid (B), TCA cycle (C), and fatty acid (D). Red dots indicate a positive correlation between gene expression, and blue dots indicate a negative correlation. ACC, adrenocortical carcinoma; BLCA, bladder urothelial carcinoma; BRCA, breast invasive carcinoma; CESC, cervical squamous cell carcinoma; CHOL, cholangiocarcinoma; COAD, colon adenocarcinoma; DLBC, diffuse large B cell lymphoma; ESCA, esophageal carcinoma; GBM, glioblastoma; HNSC, head and neck squamous cell carcinoma; KICH, kidney chromophobe; KIRC, kidney renal clear cell carcinoma; KIRP, kidney renal papillary cell carcinoma; LAML, acute myeloid leukemia; LGG, lower grade glioma; LIHC, liver hepatocellular carcinoma; LUAD, lung adenocarcinoma; LUSC, lung squamous cell carcinoma; MESO, mesothelioma; OV, ovarian serous cystadenocarcinoma; PAAD, pancreatic adenocarcinoma; PCPG, pheochromocytoma and paraganglioma; PRAD, prostate adenocarcinoma; READ, rectum adenocarcinoma; SARC, sarcoma; SKCM, skin cutaneous melanoma; STAD, stomach adenocarcinoma; TGCT, testicular germ cell tumors; THCA, thyroid carcinoma; THYM, thymoma; UCEC, uterine corpus endometrial carcinoma; UCS, uterine carcinosarcoma; UVM, uveal melanoma.

Accumulated evidence has corroborated that metabolic reprogramming could affect the immune response and clinical prognosis in cancer patients.^29^, ^40^, ^41^, ^42^, ^43^ We examined the association between the expression of IGFBPs and four metabolic pathways (carbohydrate, amino acid, TCA cycle, and fatty acid). Most IGFBPs were positively associated with carbohydrates (Figure 5A). For amino acids and the TCA cycle, IGFBP5 expression had the highest negative correlation in COAD (*p* < 0.001, Figure 5B,C). Interestingly, in TGCT, a positive correlation was observed between IGFBPs and fatty acids (*p* < 0.001). With regard to carbohydrates, IGF1, IGF2, and insulin are closely related hormones that are responsible for the regulation of metabolic homeostasis, development, and growth of the organism.^44^ Due to the high homology between IGF1 and insulin, IGF1 can also maintain glucose homeostasis, although its prominent role is to exert proliferation effects.^45^ The MAPK and PI3K/AKT pathways are two known insulin resistance pathways.^46^, ^47^ Additionally, the activities of IGF1 and 2 are regulated by the differential expression of their corresponding receptors and IGFBPs. We explored the ability of IGFBPs to regulate carbohydrates through the MAPK and PI3K/AKT pathways (Figure S7). The results suggested a positive correlation between IGFBP expression and the MAPK signaling pathway (Figure S7A). The PI3K/AKT signaling pathway was heterogeneous among different tumors (Figure S7B). Notably, IGFBPs positively correlated with PI3K/AKT in OV, ACC, GBM, LGG, and UVM but negatively correlated with THYM.

### 3.6 The expression and prognosis of IGFBPs in ovarian cancer

**FIGURE 6:**
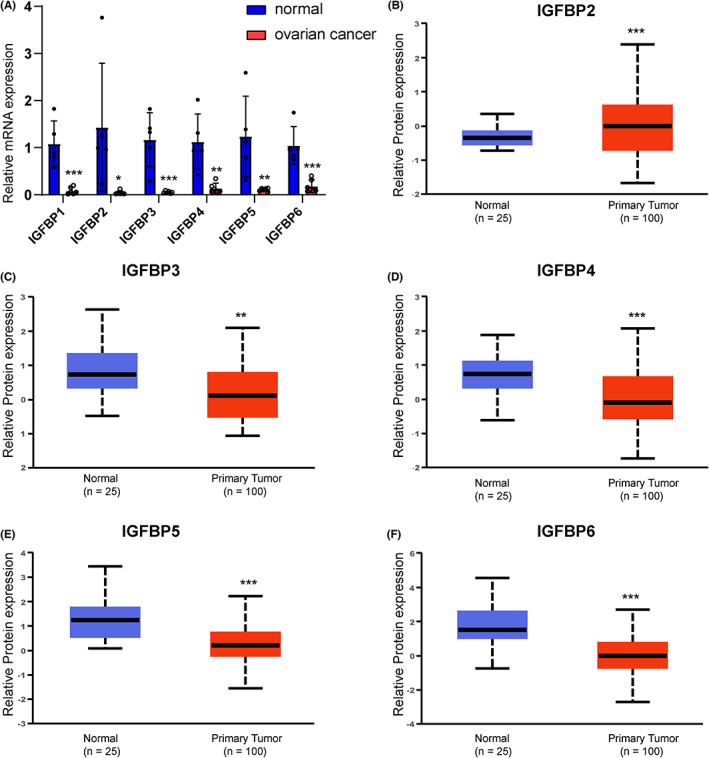
The expression of IGFBPs in ovarian cancer. (A) The mRNA expression in six ovarian cancer tissues and five normal ovarian tissues. The relative protein expression of IGFBP2 (B), IGFBP3 (C), IGFBP4 (D), IGFBP5 (E), and IGFBP6 (F) in ovarian cancer. The red and blue boxes represent tumor tissues and normal tissues, respectively. *p < 0.05, **p < 0.01, ***p < 0.001.

We know from the above results that IGFBPs are downregulated in ovarian cancer and IGFBP3/4/5/6 is positively correlated with immune scores in ovarian cancer. In addition, they are closely associated with EMT, angiogenesis, hypoxia, and carbohydrate in ovarian cancer. Therefore, we selected the patients with ovarian cancer for validation. We used PCR to study the mRNA expression level of IGFBPs, which suggested that the expression levels of IGFBPs were downregulated in ovarian cancer. Additionally, we obtained the protein expression levels of IGFBPs in the CPTAC ovarian cancer cohort. Unfortunately, data on the protein expression level of IGFBP1 in ovarian cancer are lacking. In the CPTAC cohort, IGFBP3, 4, 5, and 6 were significantly downregulated in ovarian cancers, consistent with the mRNA expression levels (*p* < 0.05, Figure 6). However, the protein level of IGFBP2 was shown to be upregulated in ovarian cancer.

**FIGURE 7:**
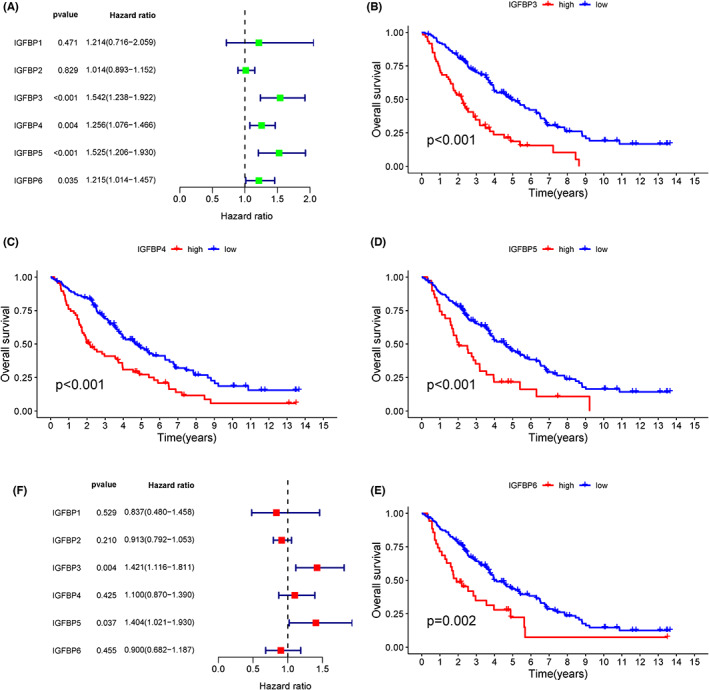
The prognostic of IGFBPs in GSE26712. (A) Univariate Cox analysis of IGFBPs in ovarian cancer. (B–E) The survival analysis of IGFBP3 (B), IGFBP4 (C), IGFBP5 (D), and IGFBP6 (E). (F) Multivariate Cox analysis of IGFBPs.

**FIGURE 8:**
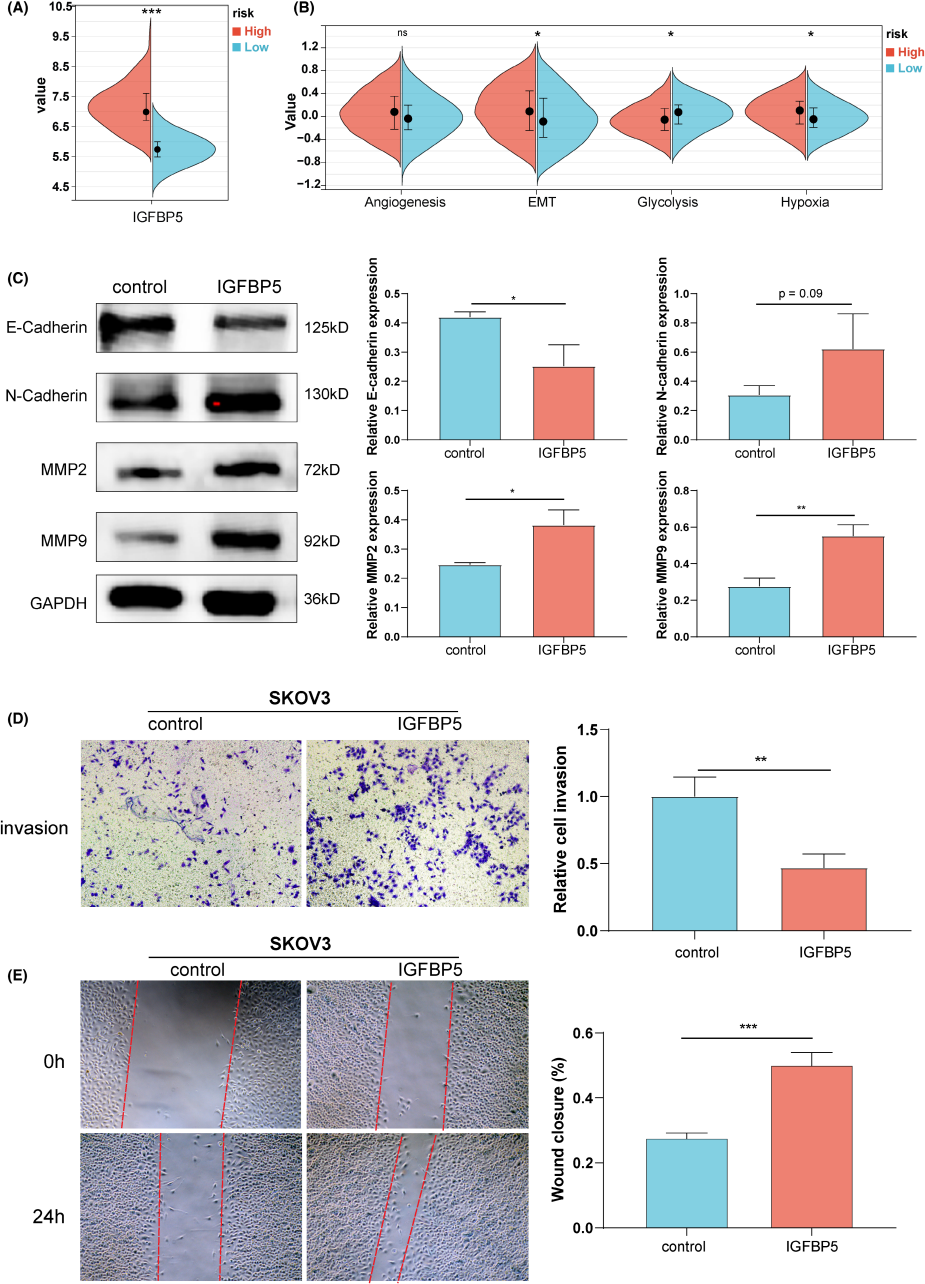
Effects of IGFBP5 on invasion and migration of ovarian cancer cells. (A) The expression of IGFBP5 in two risk groups. (B) The levels of tumor biological pathways in two risk groups. (C) Representative EMT markers were assayed by western blot. (D) A transwell invasion assay was performed to evaluate the invasion ability of SKOV3 without or with IGFBP5 recombinant protein (5 ng/mL) for 24 h. (E) A wound healing assay was performed to evaluate the migration ability of SKOV3 without or with IGFBP5 recombinant protein (5 ng/mL) for 24 h. *p < 0.05, **p < 0.01, ***p < 0.001. ns means no statistic difference.

GSE26712 was used to explore the prognostic role of IGFBPs in ovarian cancer. We performed a univariate Cox and survival analysis based on IGFBPs expression in different ovarian cancer patients and the results revealed that IGFBP3/4/5/6 was the risk factor (Figure 7A–E). After correction using multivariate analysis, we observed that IGFBP3 and IGFBP5 remained risk factors (Figure 7F). Gianuzzi et al. reported that levels of IGFBP3 were lower in patients with ovarian cancer.^48^ Moreover, IGFBP3 plays an antiapoptotic effect in ovarian cancer.^49^ Compared with IGFBP3, the studies on the relationship between IGFBP5 and ovarian cancer are limited. We have explored the expression of IGFBP5 protein in clinical samples and found that IGFBP5 was downregulated in ovarian cancer (Figure S8). Furthermore, in GSE26712, we divided ovarian cancer patients into two risk groups with a cut‐off of 6.508 based on the level of IGFBP5 (Figure 8A). Next, we compared the levels of four tumor biological pathways in two risk groups and observed that the EMT and hypoxia pathways were enriched in the high‐risk group, while glycolysis was enriched in the low‐risk group, which was consistent with the results of the above studies in pan‐cancer (Figure 8B). Exogenous administration of IGFBP5 protein showed that IGFBP5 could promote the procession of EMT in ovarian cancer cells (Figure 8C). Additionally, we verified that it promotes the invasion and migration of ovarian cancer cells (*p* < 0.05, Figure 8D,E). Furthermore, we used the GSE140082 dataset for external validation, and the results of the survival analysis were consistent with the results of GSE26712 (Figure S9). To investigate the biological significance of IGFBPs in ovarian cancer, we conducted GSEA in GSE140082 (Figure S10). The GSEA results of all IGFBPs except for that of IGFBP2 were similar. Interestingly, IGFBP1 and IGFBP4 positively regulated estrogen responses (*p* < 0.05). Specifically, IGFBP1/3/4/5/6 positively regulated EMT (*p* < 0.05), which was consistent with previous results in pan‐cancer and GSE27612.

## 4 DISCUSSION

In this article, we report the expression of IGFBPs among various cancers and analyzed the prognostic value of IGFBPs. Notably, the expression levels of IGFBPs were downregulated in both ovarian and breast cancer, whereas increased expression levels of IGFBPs were observed in GBM and PAAD. Additionally, among the four Cox proportional hazards model analyses of OS, DFI, PFI, and DSS, IGFBP1 was a risk gene in STAD. In LGG, IGFBP2 and IGFBP3 play a detrimental role. IGFBP4 was a protective factor in UCEC. The expression of IGFBP6 was associated with a longer survival time in KIRP. The results suggested that IGFBPs were differentially expressed in certain cancers and corresponding controls, which also correlated with the prognosis of patients.

Genome instability, evading immune destruction, activating invasion and metastasis, and reprogramming energy metabolism were typical hallmarks of tumors.^2^ Genomic instability leads to the accumulation of mutations in cells that can become cancerous. Mutations in genes in cancer cells lead to rapid evolution that allows them better and more quickly adapt to the TME and treatment‐induced stress. Cairns proposed that cancer is an evolutionary process of genetic instability and natural selection.^50^ Somatic mutations could be observed in 90% of cancer genes.^51^ Somatic missense mutations strongly contribute to the generation of new tumor epitopes. In this study, we analyzed the correlation between the expression of IGFBPs and TMB. Notably, IGFBP expression positively correlated with TMB in LCG, LAML, and THYM. Interestingly, the expression of IGFBPs was observed to be negatively associated with TMB in most sex‐related tumors, such as BRCA, UCEC, and OV. High TMB was associated with improved survival and response to immune checkpoint inhibitor treatments.^37^ The results suggested that the high expression of IGFBPs and concomitant low TMB in most cancers may be associated with worse prognosis in sex‐related tumors.

To evade immune destruction, weakly immunogenic cancer cells are believed to be able to grow and generate solid tumors, whereas highly immunogenic cancer cells are eliminated by the immune system.^2^ Recently, numerous studies have noted that the immune escape mechanism of tumor cells is related to their tumor microenvironment and immune invasion.^37^, ^52^, ^53^ Indeed, TME consists not only tumor cells but also many other cells related to the tumor microenvironment, such as normal stromal cells, immune cells, vascular endothelium, and blood cells within blood vessels.^54^ Immune cells are most prominent among these cells. Many types of immune cells exist, and they play different roles in the antitumor process and tumor immune escape.^55^ Tumor growth, invasion, and metastasis are all related to immune cells. The second most important cell types are stromal cells, which are also thought to play an important role in tumor growth, disease progression, and drug resistance.^56^ We analyzed the correlation between the expression of IGFBPs and immune and stromal scores. A high positive correlation was shown between IGFBP3/4/5/6 and the immune scores. Interestingly, the correlation between IGFBP3/4/5/6 and stromal scores was consistent with the trend in the immune scores.

Regarding activating invasion and metastasis, EMT is a process in which epithelial cells acquire mesenchymal morphology and can be activated temporarily or stably to varying degrees during the invasion and metastasis of cancer cells.^57^, ^58^ In tumors, angiogenesis and cancer cell invasion are tightly connected.^59^ Tumor cells can secrete vast proangiogenic factors that contribute to the formation of an abnormal vascular network. Due to the immature and permeable characteristics of tumor blood vessels, the tumor microenvironment is characterized by poor perfusion and hypoxia.^60^ In a hypoxic microenvironment, hypoxia‐inducible factor‐1α can induce the upregulation of glycolytic enzymes.^61^ Cancer cells that were able to adapt to anoxic conditions through glycolysis were screened. These cells are more invasive and aggressive and can also impede the tumor‐killing action of immune cells.^60^ Notably, the expression of IGFBPs positively correlated with EMT, angiogenesis, and hypoxia, which suggested that they may play a crucial role in tumor invasion and metastasis. The correlation between IGFBPs and glycolysis was not stronger than that with EMT, angiogenesis, or hypoxia.

Metabolic reprogramming is essential for the unlimited reproduction of cancer cells and affects the tumor immune response and clinical prognosis in cancer patients.^62^, ^63^, ^64^ We acquired four previously reported metabolic signature gene sets, including amino acid metabolism, carbohydrate metabolism, lipid metabolism, and TCA, and identified that the expression of IGFBPs was positively associated with carbohydrates in most tumors. For amino acids and TCA cycle, IGFBP5 expression had the highest negative correlation. Interestingly, in TGCT and PCPG, there was a positive correlation between IGFBPs and fatty acid. However, an inverse association was observed in THYM. The MAPK and PI3K/AKT pathways are two known insulin resistance pathways that are closely connected with carbohydrates.^65^ We explored whether IGFBPs regulate carbohydrates through the MAPK and PI3K/AKT pathways. The results suggested a positive correlation between IGFBPs expression and the MAPK signaling pathway. Notably, IGFBPs positively correlated with PI3K/AKT in OV, ACC, GBM, LGG, and UVM but negatively correlated with THYM. Furthermore, numerous studies have reported that the PI3K/AKT and MAPK pathways are closely associated with cancer invasion and metastasis.^66^, ^67^, ^68^ PI3K and MAPK signaling pathways may be involved in the biology by which IGFBPs mediate tumor invasion, migration, and metabolic reprogramming.

Finally, we analyzed the IGFBP mRNA expression in ovarian cancer samples, and the results suggested that the expression levels of IGFBPs were downregulated in ovarian cancer. Additionally, the protein expression levels of IGFBPs in the CPTAC cohort were consistent with the mRNA expression levels, except for the expression of IGFBP2. Subsequently, GSE26712 was employed to validate the results of the previous pan‐cancer analysis. The results revealed that IGFBP3/4/5/6 was the risk factor in OC. After correction using multivariate analysis, we observed that IGFBP3 and IGFBP5 remained risk factors. Compared with IGFBP3, the studies on the relationship between IGFBP5 and ovarian cancer are limited. In GSE26712, we divided ovarian cancer patients into two risk groups based on the expression of IGFBP5. Next, we compared the levels of four tumor biological pathways in two risk groups and observed that the EMT and hypoxia pathways were enriched in the high‐risk group, while glycolysis was enriched in the low‐risk group. Additionally, IGFBP5 was proved which promotes the invasion and migration of ovarian cancer cells. Furthermore, we used the GSE140082 dataset for external validation, and the results were consistent with the results of GSE26712 and pan‐cancer.

In summary, the pan‐cancer analysis examined the expression of IGFBPs and their prognostic value in various cancers. Next, the associations between four typical hallmarks of tumors and IGFBPs were comprehensively analyzed. Our data suggested that IGFBP5 can serve as an independent prognostic factor in ovarian cancer. However, some limitations remained. First, we matched each solid cancer type in TCGA with the closest corresponding healthy tissue in the GTEx. Because the source of patients and specific information were not clear, this comparison is subject to bias. Second, this study mostly focused on bioinformatic analysis, and some correlations described herein are only suggestive. Therefore, the mechanism by which IGFBPs specifically affect the tumor progression requires additional in vitro and in vivo experiments.

## 5 CONCLUSIONS

Additional knowledge of the complex regulatory mechanisms of IGFBPs is essential to better exploit its potential clinical utility for diagnostic and therapeutic purposes. This study revealed that IGFBPs are closely correlated with tumor invasion and metastasis. Additionally, IGFBPs may regulate the metabolic reprogramming to mediate tumor carcinogenesis. The PI3K and MAPK signaling pathways may be involved in the biology by which IGFBPs mediate tumor invasion, migration, and carbohydrate production. Our results could provide underlying targets for the design of laboratory experiments to elucidate the mechanism of IGFBPs in cancers and identify IGFBP5 as a prognostic factor in ovarian cancers. Further studies focusing on the mechanisms of IGFBPs at both the cellular and molecular levels, especially investigating the mechanisms underlying positive correlations, will be beneficial to clarify the role of IGFBPs among cancers.

## AUTHOR CONTRIBUTIONS

**Wei Tan:** Conceptualization (lead); writing – original draft (lead). **Jie Zhang:** Investigation (equal); resources (equal). **Zhimin Deng:** Methodology (equal); validation (equal). **Fangfang Dai:** Data curation (equal); visualization (equal). **Lujia Tang:** Formal analysis (equal); software (equal). **Wei Hu:** Funding acquisition (equal); writing – review and editing (equal). **Hua Liu:** Funding acquisition (lead); writing – review and editing (equal).

## FUNDING INFORMATION

This work was supported by cross‐innovation talent project in Renmin Hospital of Wuhan University (Grant No. JCRCZN‐2022‐016); the Natural Science Foundation of Hubei Province (Grant No. 2022CFB252); the Undergraduate education quality construction comprehensive reform project (Grant No. 2022ZG282); and Innovative Research Group Project of the National Natural Science Foundation of China (Grant No. 82071655).

## CONFLICT OF INTEREST STATEMENT

The authors declare no competing interests.

## ETHICS STATEMENT

This study was approved by the Ethics Committee of the Renmin Hospital of Wuhan University and carried out following the Declaration of Helsinki.

## Supporting information


Figure S1.

Table S1.

## Data Availability

The data that support the findings of this study are available in the supplementary material of this article.
