# Supplementary material for: Pan‐cancer analysis of oncogenic role of insulin‐like growth factor‐binding proteins and validation in ovarian cancer
Source: Cancer Med. 2023 May 18;12(13):14833–50. doi: 10.1002/cam4.6073 (PMC10358252; doi:10.1002/cam4.6073)
Supplement: Supplementary file 1 — Figure S1. [file CAM4-12-14833-s002.docx]

**Supplementary Figures**

**
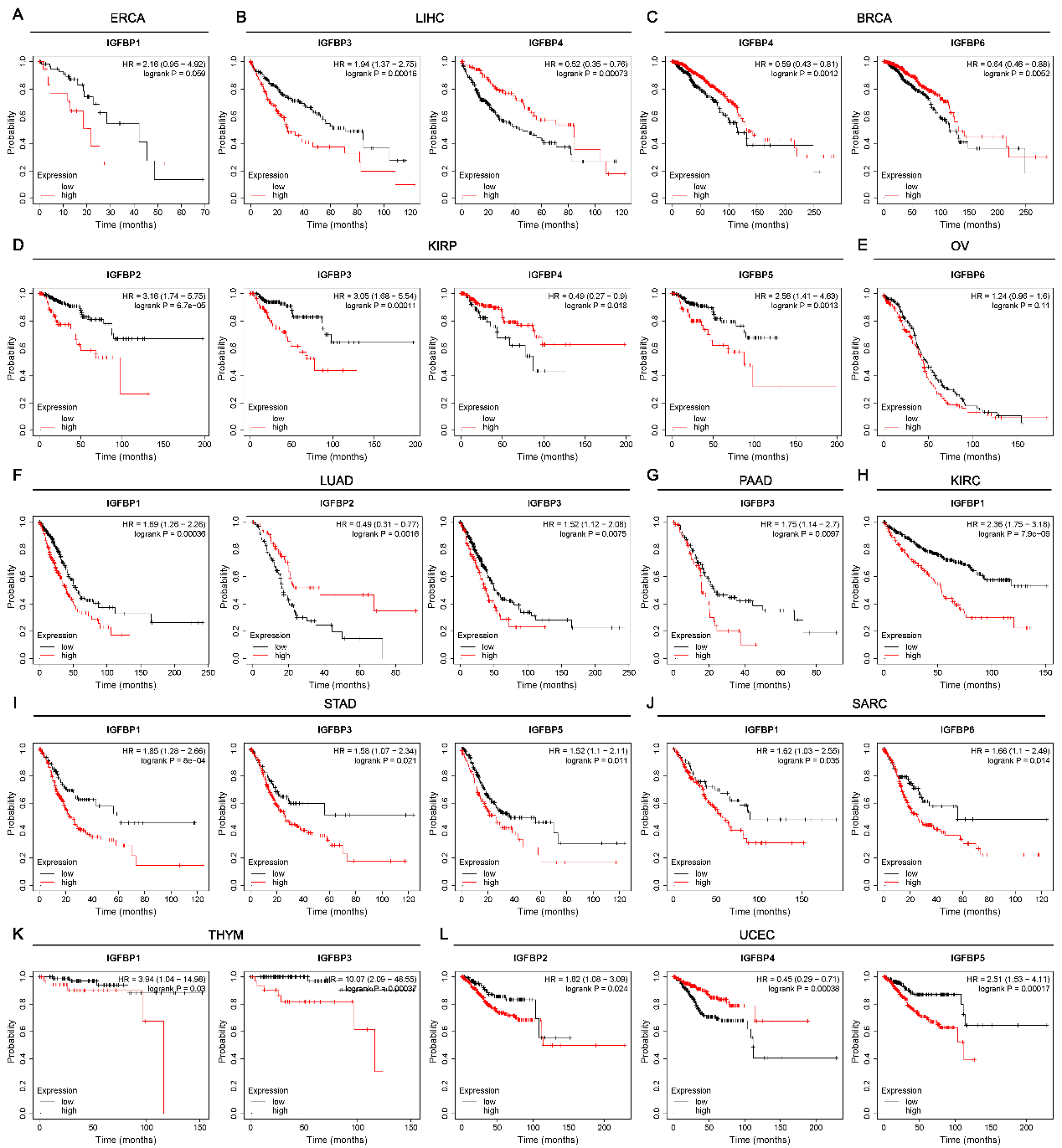
**

**Figure S1.** OS curves comparing the high and low expression of IGFBPs in Kaplan-Meier Plotter database. (A) IGFBP1 in ERCA, (B) IGFBP3 and IGFBP4 in LIHC, (C) IGFBP4 and IGFBP6 in BRCA, (D) IGFBP2, IGFBP3, IGFBP4 and IGFBP5 in KIRP, (E) IGFBP6 in OV, (F) IGFBP1, IGFBP2 and IGFBP3 in LUAD, (G) IGFBP3 in PAAD, (H) IGFBP1 in KIRC, (I) IGFBP1, IGFBP3 and IGFBP5 in STAD, (J) IGFBP1 and IGFBP6 in SARC, (K) IGFBP1 and IGFBP3 in THYM, (L) IGFBP2, IGFBP4 and IGFBP5 in UCEC.

**
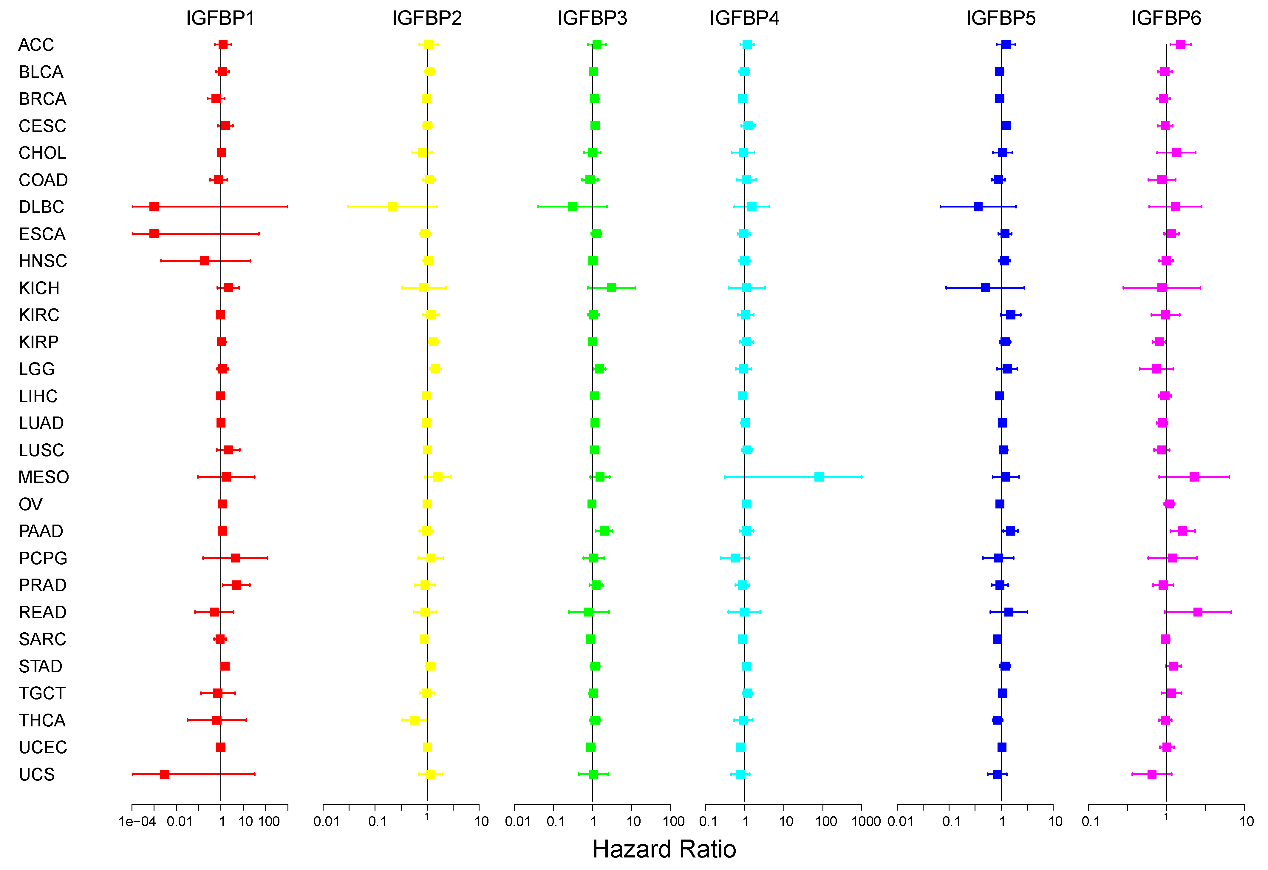
**

**Figure S2.** Association between IGFBPs expression and disease-free interval (DFI). Forest plot of DFI association with IGFBPs in 33 tumor types. Different colored lines indicate the risk value of different genes in tumors, hazard ratio < 1 represent low risk and hazard ratio > 1 represent high risk.


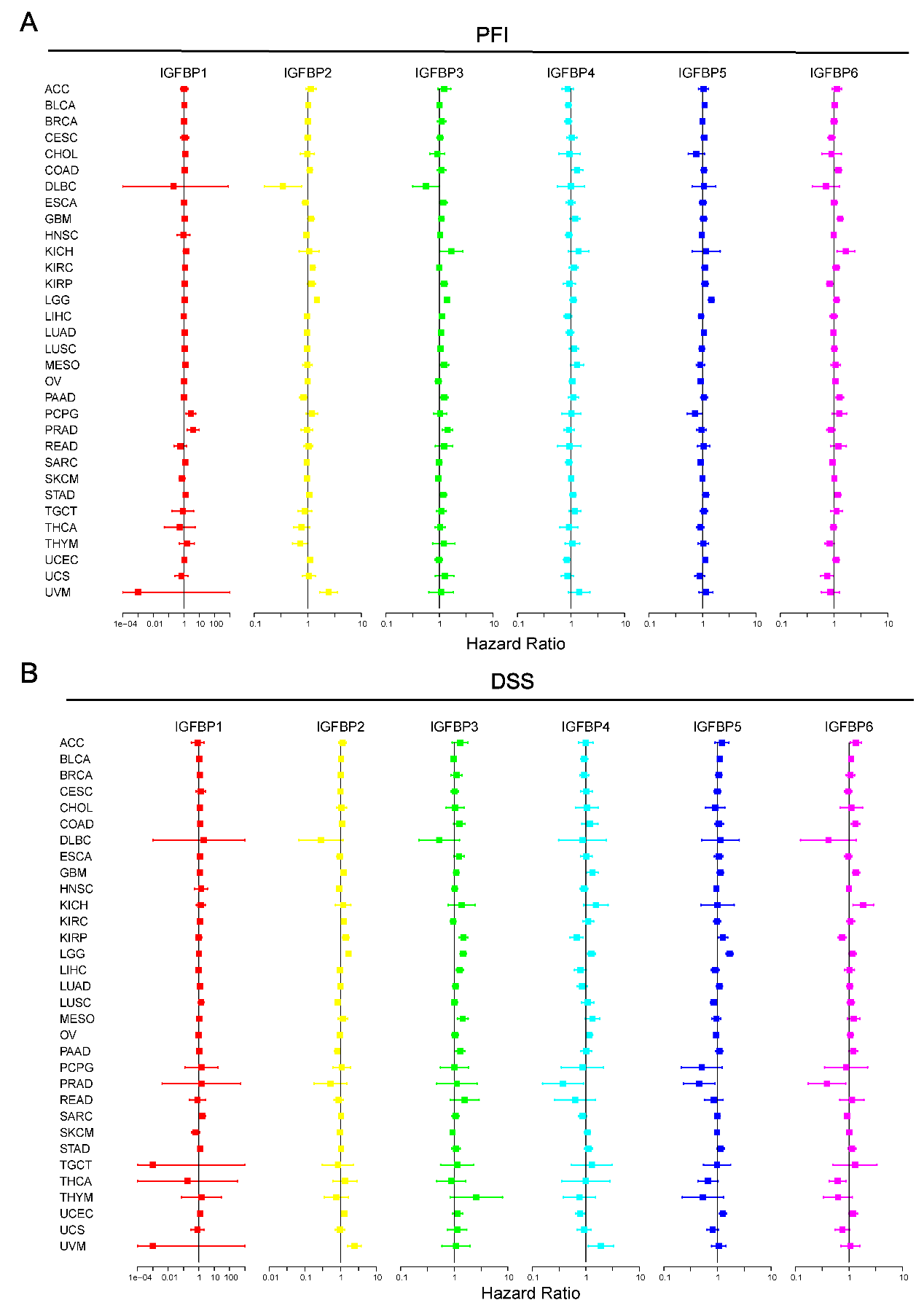


**Figure S3.** Association between IGFBPs expression and progression-free interval (PFI), disease-specific survival (DSS). Forest plot of PFI (A) and DSS (B) association with IGFBPs. Different colored lines indicate the risk value of different genes in tumors, hazard ratio < 1 represent low risk and hazard ratio > 1 represent high risk.


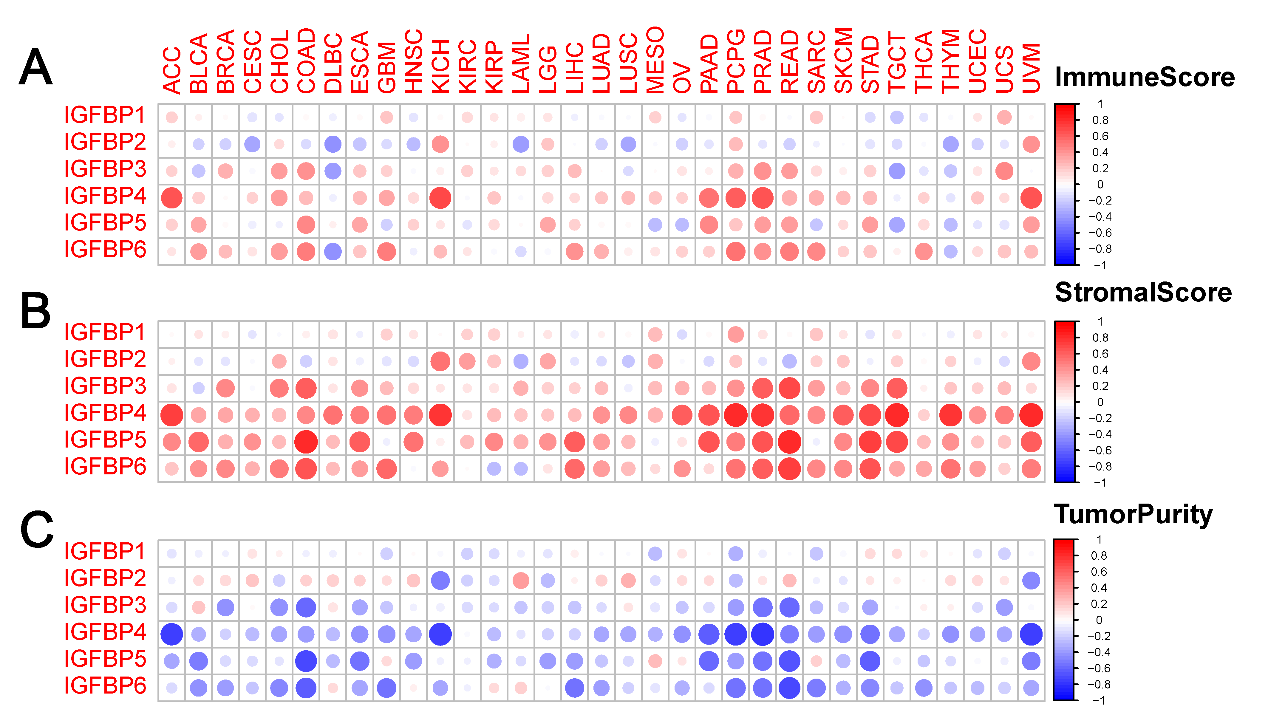


**Figure S4.** The association of between the expression of IGFBPs and tumor immune microenvironment. The heatmap of the correlation of immune score (A), stromal score

(B), and tumor purity (C) with IGFBPs. Red dots indicate a positive correlation between

gene expression, and blue dots indicate a negative correlation.


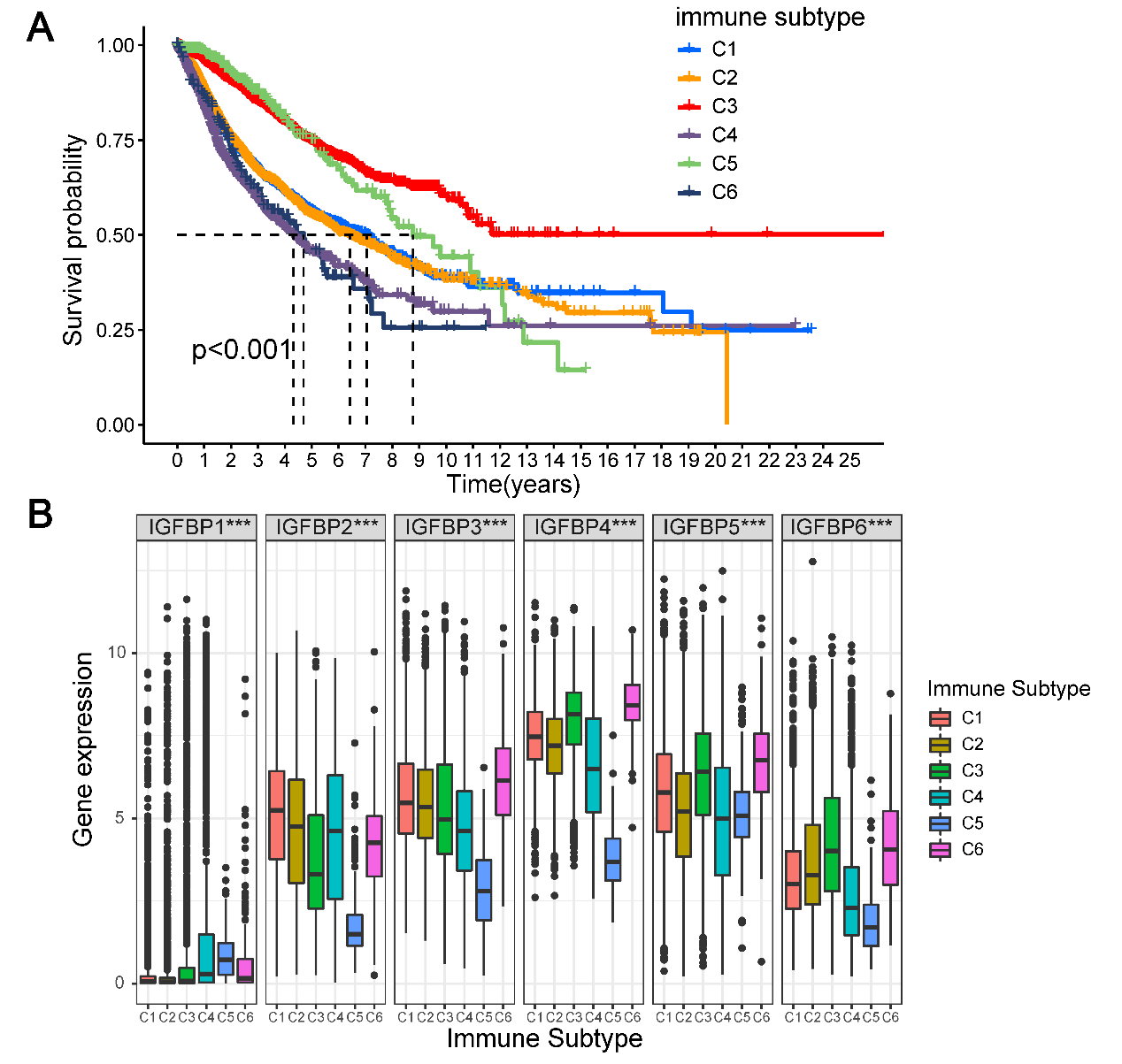


**Figure S5.** Association of IGF family expression with immune infiltrate subtypes across the cancer types. (A) The survival analysis of six immune-subtypes. (B) The expression of IGFBPs in six immune subtypes. ****p* < 0.001.


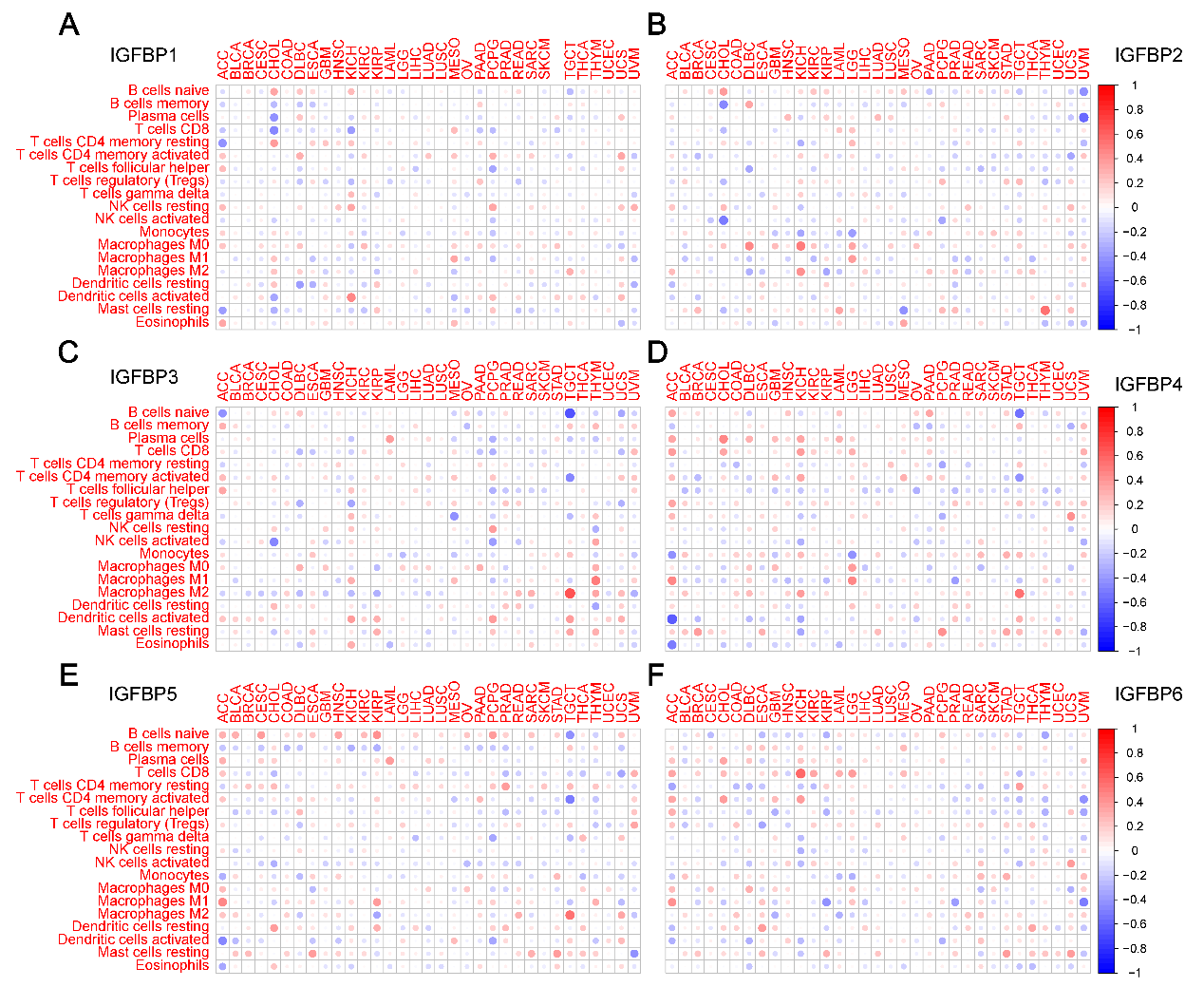


**Figure S6.** The immune landscape of IGFBPs across 33 cancers. (A-F) Correlation of IGFBP1 (A), IGFBP2 (B), IGFBP3 (C), IGFBP4 (D), IGFBP5 (E), and IGFBP6 (F) expression with immune infiltration level. Red dots indicate a positive correlation between gene expression, and blue dots indicate a negative correlation


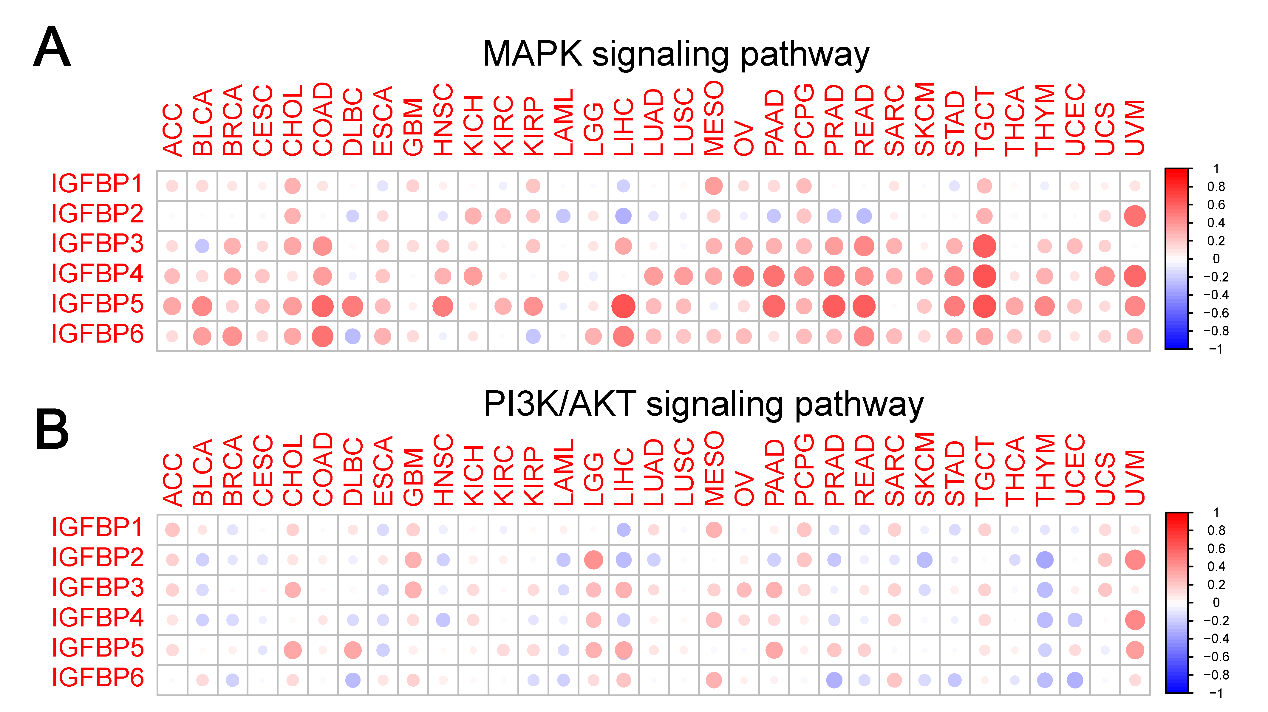


**Figure S7.** Association of IGFBPs expression with insulin resistance-related signaling pathway. (A-B) Correlation matrix between IGFBPs and MAPK signaling pathway (A) and PI3K/AKT signaling pathway (B).


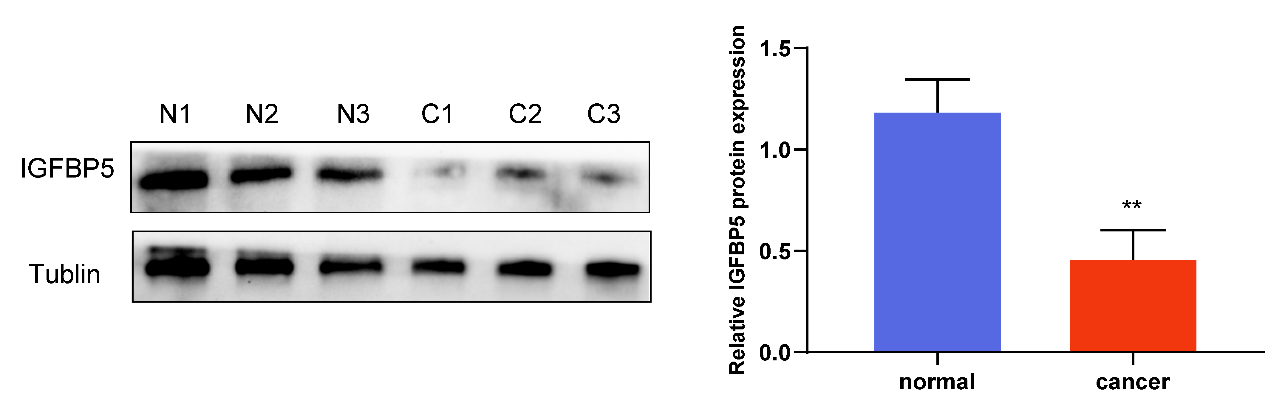


**Figure S8.** The expression of IGFBP5 in clinical samples. **p < 0.01.


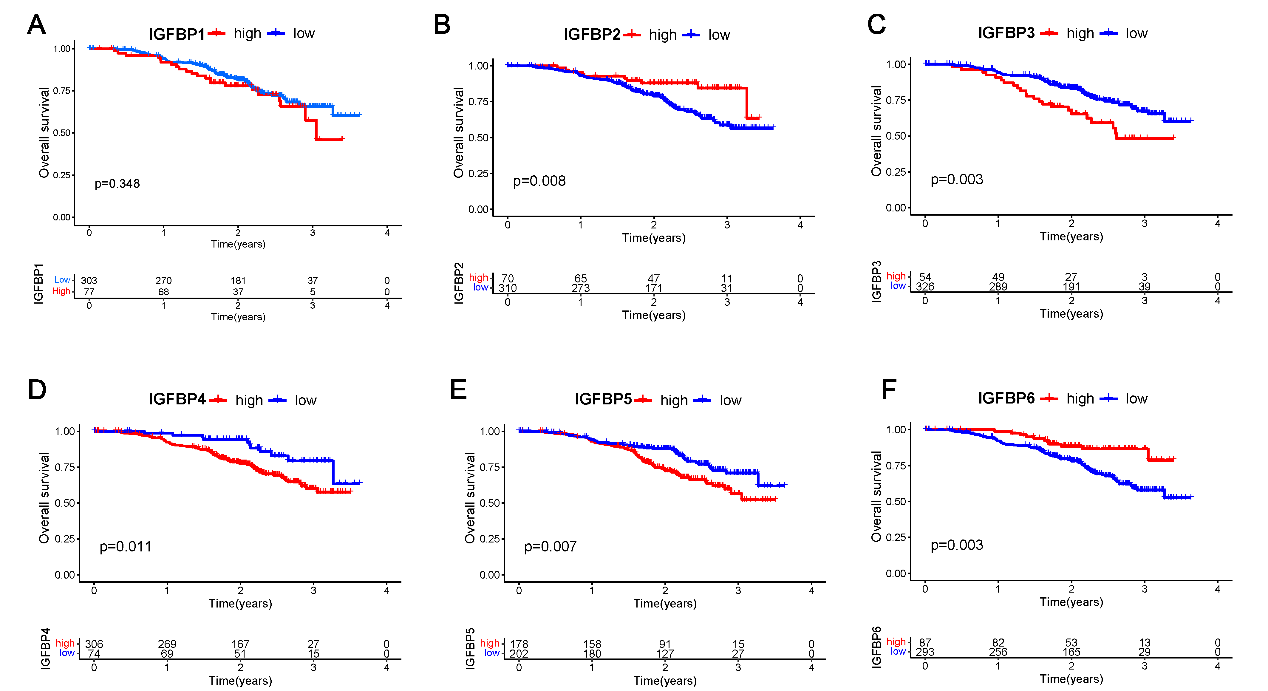


**Figure S9.** The survival analysis of IGFBP1 (A), IGFBP2 (B), IGFBP3 (C), IGFBP4 (D), IGFBP5 (E) and IGFBP6 (F) in GSE140082.


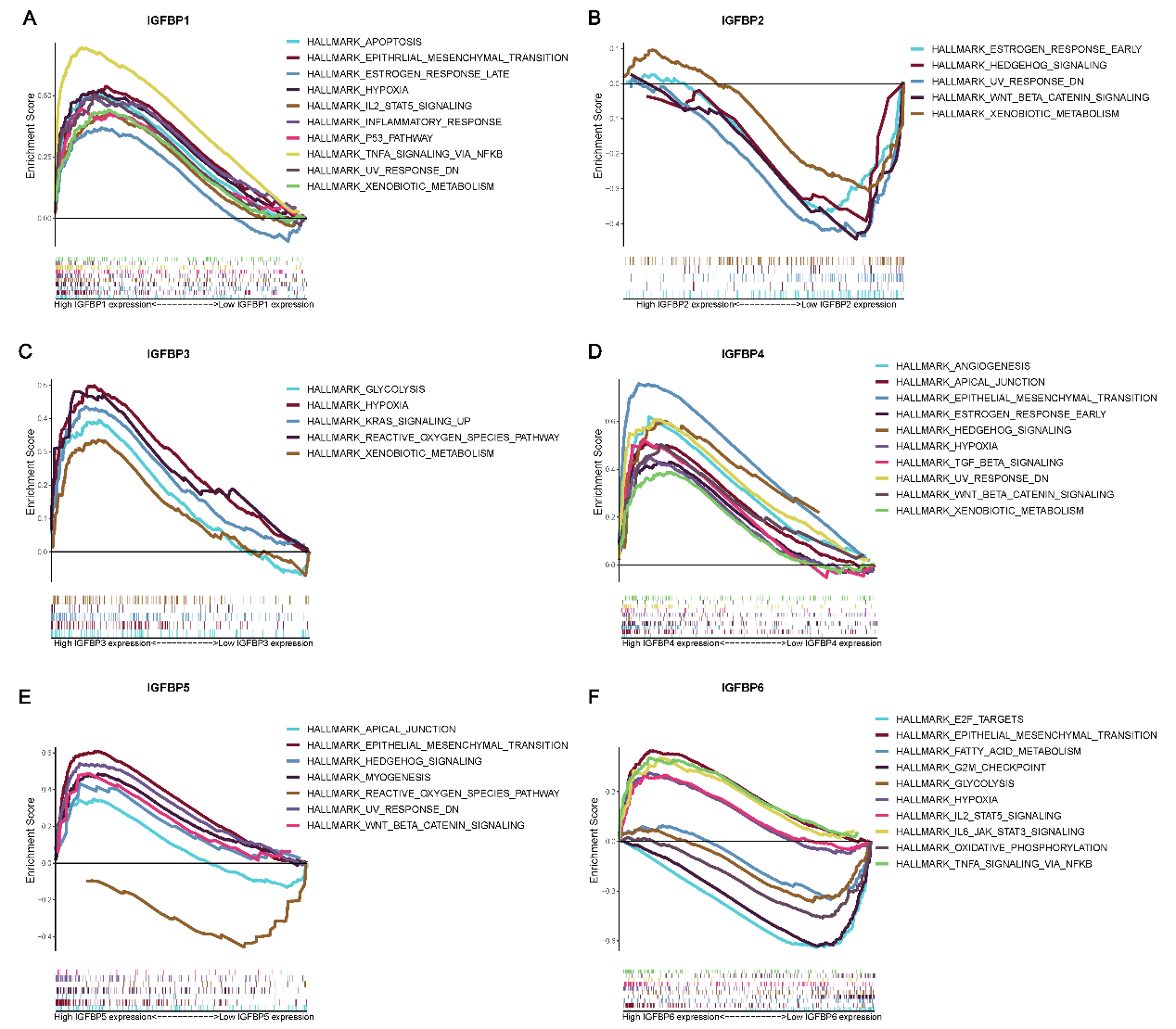


**Figure S10.** GSEA of top functional terms associated with IGFBPs in ovarian cancer. GSEA of IGFBP1 (A), IGFBP2 (B), IGFBP3 (C), IGFBP4 (D), IGFBP5 (E), and IGFBP6(F) based on HALLMARK gene sets.
